# Diagnosing Sepsis and Identifying Bacterial Resistance Genes Directly from Whole Blood Using a Rapid Real-Time PCR System

**DOI:** 10.3390/ijms27177669

**Published:** 2026-08-27

**Authors:** Selbi Begmyradova, Lütfiye Öksüz, Zerrin Aktas, Oral Öncül

**Affiliations:** 1 Department of Infectious Diseases and Clinical Microbiology, Istanbul Faculty of Medicine, 34093 Istanbul, Türkiye; selbi.begmyradowa@gmail.com; 2 Department of Medical Microbiology, Istanbul Faculty of Medicine, 34093 Istanbul, Türkiye; zaktas@istanbul.edu.tr (Z.A.); oraloncul@istanbul.edu.tr (O.Ö.)

**Keywords:** sepsis, rapid diagnosis, whole blood, blood culture, rapid real-time-PCR

## Abstract

Rapid diagnosis is vital for patients with sepsis. Therefore, this study was conducted to assess the diagnostic performance of rapid Real-Time PCR (rRT-PCR), which detects 32 pathogens and common resistance genes in whole blood within four hours. This study included 160 adult patients with sepsis. Whole-blood samples and BCs were collected from all patients simultaneously, and causative microorganisms were investigated using an rRT-PCR panel and an automated BC system. The panel was also used to detect common resistance genes. The microorganisms most frequently isolated from the BCs were *Staphylococcus aureus*, *Klebsiella pneumoniae*, and *Escherichia coli*. Among the resistance genes (7.5%) detected by rRT-PCR, *mec*A, OXA-48, and CTX-M were the most frequently identified. The agreement rate between the BC and rRT-PCR results was statistically significant at 79.4%. In the concordance group, the mean age was higher, while the duration of positive BC signals was significantly shorter. Additionally, the sensitivity and specificity of the rRT-PCR method were found to be 80.7% and 99.0%, respectively. Thus, the panel’s ability to detect the causative bacteria and common resistance genes within four hours offers a significant clinical advantage. This allows for early diagnosis in patients with sepsis, prevents progression to septic shock, and reduces mortality rates.

## 1. Introduction

Sepsis is a serious clinical condition in which infection is combined with a systemic inflammatory response syndrome, leading to multiple-organ dysfunction and high mortality rates [1,2]. According to the Global Burden of Sepsis study, approximately 49 million cases of sepsis occur worldwide each year, and about 11 million people die from sepsis [2,3]. Early diagnosis and appropriate antimicrobial treatment are crucial for reducing mortality, but identifying the correct causative agent and initiating the appropriate antibiotic in patients with sepsis are often difficult [1]. This is due to the inadequacy of current diagnostic methods, especially in terms of time and their low sensitivity [4,5].

The “gold standard” blood culture (BC) method has disadvantages, such as long lead times, false negatives, and problems in detecting rare or difficult-to-cultivate pathogens, leading to prolonged empirical treatment and increased mortality, as well as paving the way for antimicrobial resistance to develop [1,4]. In recent years, real-time polymerase chain reaction (Real-Time PCR)-based systems have been able to detect the genetic material of microorganisms directly from clinical samples, provide results in a very short amount of time, enable the initiation of targeted antibiotic therapy, and identify the causative agent, even in patients who have already started antibiotics [1,5]. The advantages of Real-Time PCR systems include their ability to detect multiple pathogens (such as Gram-positive and Gram-negative bacteria and yeast species) in a single test, as well as identify antimicrobial resistance genes (e.g., *mec*A, *van*A, and *bla*CTX-M), thus enabling more informed guidance of empirical treatment [6].

This study aimed to investigate the effectiveness of the rapid RT-PCR (rRT-PCR) system on whole blood samples from patients diagnosed with sepsis and to compare the results obtained with conventional BC methods. Within the scope of this study, the positivity rates of both methods, the distribution of causative microorganisms, the difference in identification time and their relationship with clinical parameters were evaluated. In addition, the clinical significance of microorganisms detected by the rRT-PCR system, the potential for contamination, and their contribution to clinical decision-making processes were analyzed.

## 2. Results

### 2.1. Patients

The mean age of the patients was 59.1 ± 17.8; fifty (31.3%) were women and the rest (68.2%) were men. The demographic, clinical, and laboratory characteristics of the patient population are shown in Table 1.

### 2.2. Blood Culture Results

Bacterial growth was detected in BCs in 54 (33.7%) patients. Of these, 51 (31.8%) showed significant results supporting the diagnosis, while three (1.8%) showed bacteria that were considered contaminants. The most frequent isolated bacteria were *Staphylococcus aureus *(6.3%), extended spectrum-beta lactamase (ESBL)-producing *Klebsiella pneumoniae* (3.8%), and ESBL-negative *Escherichia coli* (3.1%). A full agreement between the BC and rRT-PCR methods was observed in 127 (79.4%, *p* = 0.000) patients with sepsis (Table 2). The mean age was significantly higher in the group with overall agreement (OA) between the BC and rRT-PCR results (60.7 ± 17.4 vs. 53.0 ± 18.2; *p* = 0.029). Additionally, no significant differences were found between the groups in terms of gender distribution, comorbidity rate, and ASA, SIRS, APACHE, and SOFA scores (*p* > 0.05). The positive signal time for BC was significantly shorter in the OA group (6.4 ± 14.8 h vs. 26.4 ± 18.1 h; *p* < 0.001) (Table 3).

### 2.3. rRT-PCR Results

According to the rRT-PCR results, a positive result indicating the presence of any microorganism was observed in 41 (25.6%) cases. In contrast, rRT-PCR results were negative in 119 (74.4%) cases. Table 4 shows the concordance between the microorganisms detected by BC and rRT-PCR, with the most frequently detected pathogens by rRT-PCR being *Staphylococcus* species in nine cases (5.6%), ESBL-producing *K. pneumoniae* in six cases (3.8%), and ESBL-negative *K. pneumoniae* in five cases (3.1%) (Table 2).

### 2.4. BC and rRT-PCR Concordance

Table 3 shows the comparison of demographic characteristics, comorbidities, and clinical severity scores according to BC and rRT-PCR concordance (Cohen’s Kappa 79.4%, *p* = 0.000, 95% CI).

In five patients with positive rRT-PCR results but negative BC results, the causative agents were isolated from clinical samples other than BC (pleural fluid, abdominal abscess, tracheal aspirate, urine, and CVP catheter).

Table 5 shows the comparison of microbiological results according to BC-rRT-PCR concordance. Upon comparing groups without and with compatible BC and rRT-PCR results, no significant differences were found in HGB, HCT, CRP, PCT, AST, bilirubin, and lactate values.

### 2.5. Mortality

Mortality was observed in 46 (28.75%) patients, and the rate of hypertension was found to be higher in the group of patients who developed mortality compared to the group that did not develop mortality (39.1% vs. 20%, *p* = 0.009) (Table 6).

In the 30-day mortality group, the ASA (*p* = 0.005), SIRS (5.2 ± 2.1 vs. 4.1 ± 2.2; *p* = 0.001), APACHE (18.9 ± 8.7 vs. 12.6 ± 7.4; *p* < 0.001), and SOFA (6.7 ± 3.9 vs. 4.4 ± 3.1; *p* < 0.001) scores, as well as ICU admission rate (80.4% vs. 54.4%; *p* = 0.007), were significantly higher than those in the group with no 30-day mortality (*p* < 0.05) (Table 6). The rate of subsequent antibiotic use was significantly higher in the group with 30-day mortality than in the group without 30-day mortality (*p* = 0.002) (Table 7). Additionally, the rates of PCT, bilirubin, and AST levels were found to be higher in the group who developed mortality compared to the no-mortality group (*p* < 0.05) (Table 8). The diagnostic performance (sensitivity, specificity, accuracy, PPV, and NPV) of the rRT-PCR method compared to the reference BC method is indicated in Table 9.

## 3. Discussion

Timely initiation of appropriate antimicrobial therapy is crucial for survival in patients with sepsis, as even a one-hour delay in effective antimicrobial treatment can significantly increase mortality. Therefore, rapid molecular diagnostic methods can provide clinical benefits by yielding microbiological results earlier than traditional culture methods [2,7]. Because delays in effective antimicrobial therapy are associated with poorer outcomes in sepsis, earlier availability of pathogen and resistance-marker information may support more timely reassessment and optimization of empirical treatment. The evaluated automated extraction and multiplex qPCR-based workflow enables direct detection from whole blood without the need to wait for microbial growth, thereby potentially shortening the time to preliminary pathogen identification [8,9].

A relevant advantage of the rRT-PCR kit is its ability to detect a broad range of bacterial and fungal pathogens concurrently, along with clinically important antimicrobial resistance markers, in the same diagnostic workflow. This is particularly important in critically ill patients, in whom polymicrobial bloodstream infections, candidemia, unexpected pathogens, or MDR organisms may complicate empirical treatment [8,10]. The simultaneous availability of pathogen and resistance-marker information, including markers associated with carbapenemase production, ESBL, and methicillin, vancomycin, and colistin resistance, may provide an early indication of potentially inappropriate empirical therapy before conventional identification and phenotypic susceptibility testing results become available [11]. As with direct-from-blood rRT-PCR-based diagnostic methods in general, appropriate implementation requires trained personnel, standardized workflow practices, strict contamination control, and careful interpretation of discrepant results [8,12]. In the present study, both rRT-PCR-positive/BC-negative and BC-positive/rRT-PCR-negative results were observed. Possible reasons for the discrepancies between the BC and rRT-PCR results include prior antimicrobial exposure, which can reduce pathogen recovery by culture, as well as the high analytical sensitivity of PCR for detecting low-level microbial DNA. Another reason is the isolation of bacteria from other samples (pleural fluid, abdominal abscess, tracheal aspirate, urine, and CVP catheter) than BC in five patients. This may be the reason for the decreased sensitivity of the rRT-PCR test compared to the reference test. In addition, pre-analytical contamination during blood sampling or sample processing cannot be excluded, particularly for skin-associated bacteria. Conversely, BC-positive/qPCR-negative results may be related to a low pathogen burden, an uneven distribution of microorganisms between paired specimens, PCR inhibition, or organisms not covered by the assay panel. Therefore, discrepant results should be interpreted cautiously in combination with clinical findings and conventional microbiological results. Prior to introducing the rRT-PCR panel into routine clinical practice, each laboratory needs to review its own financial situation, potential challenges in laboratory workflow, technical requirements, and the need for adequately trained personnel. Although the initial costs of molecular testing may be higher than those of conventional methods, the rapid availability of results may facilitate earlier pathogen identification and optimization of antimicrobial therapy. Therefore, following appropriate planning and implementation, the panel may contribute to antimicrobial stewardship strategies by supporting timely targeted treatment and reducing unnecessary broad-spectrum antibiotic use.

Positive BC but negative rRT-PCR results may be due to the presence of a small number of microorganisms in whole blood, as the volume of blood collected in BC vials is larger and the BC vials contain a rich culture medium and are incubated at 37 °C for at least 24–48 h. Additionally, rare pathogens not covered by the molecular panel (*Morganella morganii*, *Haemophilus influenzae*, *Candida parapsilosis*, and *Cryptococcus* spp.) may have also led to negative rRT-PCR results. This issue highlights areas for improvement in the panel. On the other hand, positive rRT-PCR but negative BC results may be due to the high analytical sensitivity of the rRT-PCR panel, which can even detect low levels of microorganism DNA. Primary infections leading to bloodstream infections (such as urosepsis, pneumosepsis, and catheter infection) also point to this result. To support this, bacteria detected by rRT-PCR in four patients (*E. coli*, *S. pneumoniae*, *C. parapsislosis*, and *Cryptococcus* spp.) were isolated from urine, pleural fluid, tracheal aspirate, abdominal abscesses, and CVP catheters. Detecting the causative pathogen within four hours using rRT-PCR can help rapidly organize the antimicrobial administration process. This clinical benefit is predicted to be life-saving in patients with Gram-negative sepsis, especially those with fungemia.

Rapid RT-PCR performed directly from whole blood showed a high concordance with the reference blood culture (BC) method (79.4%), supporting its reliability as a rapid diagnostic tool for sepsis. This agreement was higher than that reported in a study from Vietnam (42%) [13] and compared favorably with other published reports [14,15,16]. The high sensitivity (80.7%) and specificity (99.0%) observed in our study further support the clinical utility of this assay. Moreover, differences among studies may be explained by variations in patient populations, PCR panels, sample types, and pre-analytical procedures [16,17,18,19,20,21].

The rapid identification of pathogens is particularly important in critically ill patients. Although 97.5% of our patients had at least one comorbidity and more than one-third had malignancy, complete concordance between BC and rRT-PCR was not associated with 30-day mortality. Instead, mortality was associated with higher SIRS, APACHE II, SOFA, and ASA scores; ICU admission; hypertension; and elevated PCT, bilirubin, and AST levels, indicating that disease severity and organ dysfunction remain the primary determinants of prognosis, consistent with the Sepsis-3 definition [22].

Antimicrobial resistance remains a major challenge in sepsis management [2]. In our cohort, resistance genes were detected in 7.5% of cases. The identification of resistance genes such as OXA-48, CTX-M, *mec*A, and ESBL within approximately four hours represents a considerable advantage over conventional susceptibility testing, which generally requires 48–72 h [23]. However, molecular detection of resistance genes was not associated with mortality, likely owing to the limited number of resistant isolates and the widespread use of broad-spectrum antibiotics.

Blood culture remains the standard and most widely used method for sepsis diagnosis worldwide, whereas rRT-PCR assays are mainly implemented in selected centers as complementary diagnostic tools due to cost and infrastructure requirements. Previous studies have compared BC- vs. PCR-based assays for sepsis diagnosis [14,16,17,18,19,20,21]. Bloos et al. emphasized that molecular assays should complement rather than replace BC [14]. Similarly, Ferrara et al. reported a lower agreement (57%) but a high NPV using the same rRT-PCR system [15]. Erdogan et al. found high specificity but lower sensitivity for direct whole-blood PCR than for PCR performed from BC bottles [17], whereas Nieman et al. reported sensitivity and specificity scores of 66.7% and 94.4%, respectively [18]. In Italy, the same rRT-PCR system achieved an overall agreement of 78% [16], similar to our study, while Rowther et al. highlighted the rapid turnaround time but variable specificity of PCR assays [19]. Likewise, an Indonesian study reported sensitivity and specificity scores of 89% and 87%, respectively [20]. In contrast, a neonatal sepsis study from Turkiye reported considerably lower sensitivity but similar specificity and NPV compared with our findings [21]. Overall, the relatively high concordance observed in our study may be related to differences in patient selection, assay characteristics, sampling time, and pre-analytical optimization. Comparative studies conducted since the introduction of molecular methods in sepsis diagnosis were examined. The overall agreement rate between molecular methods and the reference BC method varied between 79 and 90%, while sensitivity and specificity rates ranged from 20 to 77% and from 65 to 100%, respectively. Additionally, the time needed to detect the causative agent ranged from 4 to 18 h.

Most patients in our cohort had received antibiotics before referral, which may have reduced BC positivity while allowing pathogen DNA to remain detectable by rRT-PCR. This finding supports the complementary role of molecular testing alongside conventional culture. Overall, the high agreement, sensitivity, and specificity observed in our study indicate that direct whole-blood rRT-PCR can provide rapid and reliable microbiological information, facilitating earlier targeted antimicrobial therapy and supporting antimicrobial stewardship in patients with sepsis.

### Limitations

A major strength of this study is its prospective design, with systematic data collection and clinical score assessment enhancing the reliability of the findings. However, the single-center design and relatively small sample size may limit the generalizability of the results. In addition, the diversity of resistance genotypes was restricted to those prevalent at our center, limiting the evaluation of the rRT-PCR assay against less common resistance mechanisms. Previous antibiotic exposure in many patients may also have reduced culture positivity and affected the agreement between BC and rRT-PCR results. Our institution is a tertiary university hospital, and patients are generally referred to our center after receiving antimicrobial treatment at other centers. It is known that prior antibiotic use negatively affects culture positivity, and pathogens present in the bloodstream cannot be isolated. Similarly, it can be assumed that pathogens whose levels in the blood decrease with antibiotic use are reduced to undetectable levels by rRT-PCR.

Ours is the first large-scale prospective study in which the rRT-PCR panel was tested. It was deemed inappropriate to intervene in the antimicrobial treatment process without first evaluating the effectiveness of the test at this stage. Since it is clear that rapid diagnosis in patients with sepsis will enable earlier targeted treatment, a new study has been planned in which treatment will be adjusted according to the rRT-PCR result.

Finally, it is likely that the absence of some uncommon pathogens in the rRT-PCR panel has contributed to its lower sensitivity, as the utility of this panel is restricted to the pathogens and resistance markers included in it. Furthermore, this panel is designed to provide complementary diagnostic support, rather than replace, conventional BCs necessary for organism detection, complete antimicrobial susceptibility testing, and epidemiological surveillance. Despite these limitations, the assay demonstrated high sensitivity, specificity, accuracy, and predictive values, supporting the reliability of the rRT-PCR method.

## 4. Materials and Methods

### 4.1. Definitions

Patients were diagnosed with sepsis based on clinical findings, laboratory parameters, and the SOFA scoring system. Specifically, an increase in the SOFA score (≥2) in the presence of infection supports the diagnosis of sepsis [2,24].

The presence of the same species and type of bacteria or fungi in both the BC and rRT-PCR test results or, in the case of no growth in the BC, the detection of bacteria using rRT-PCR from a different clinical sample of the same patient, such as pleural fluid, sputum, joint fluid, or peritoneal fluid, was defined as “concordance between the two tests”.

The difference in bacteria detected in the BC and rRT-PCR test results or the detection of bacteria or fungi in only one BC or rRT-PCR test while not detecting them in the other is defined as “discordance between the two tests”. In one of two sets of BCs collected from two different anatomic sites, the growth of skin flora bacteria (coagulase-negative staphylococci, alpha-hemolytic streptococci, etc.) was defined as “BC contamination” [25].

### 4.2. Study Design

This single-center prospective study included 160 adult patients who presented to our hospital’s emergency departments or other wards, were suspected of having sepsis in the differential diagnosis, and were followed up by the Infectious Diseases and Clinical Microbiology Unit. Two sets of BCs and one tube of whole blood were taken from each patient for rRT-PCR.

### 4.3. Patients

This study was conducted between October 2024 and January 2026. Detailed medical histories and systemic physical examinations were performed on patients admitted to our hospital’s emergency department, wards, or Intensive Care Unit (ICU) with a preliminary diagnosis of sepsis or those who developed sepsis symptoms while in the ICU and for whom consultation was requested from the Infectious Diseases and Clinical Microbiology Department. In addition to pathological findings, all possible risk factors and patient data were recorded, and routine laboratory analyses, biochemical parameters, BCs, and rRT-PCR tests were planned. Empirical antibiotic treatment was initiated following the patient’s physical examination findings and laboratory analyses. Then, rRT-PCR test results obtained approximately four hours after whole-blood sampling and BC results obtained 24–48 h later were recorded. Empirical antibiotic treatments initiated based on the patients’ BC results were reviewed, and new treatment plans were made as needed. The obtained test results were evaluated within the context of the patients’ previously used antibiotic treatments and general condition, and data from both diagnostic methods were compared.

### 4.4. Data Collection

The following information was recorded for each patient: demographic characteristics; presenting complaints; underlying comorbidities; history of previous surgery, history of previous antibiotic use; laboratory findings (complete blood count, CRP, PCT, liver function tests, and kidney function tests); ASA, SIRS, quick SOFA, and SOFA scores; and antibiotic therapy regimens administered upon hospitalization. Furthermore, the clinical course (survival, mortality, etc.) was also recorded.

### 4.5. Blood Culture Method

Skin antisepsis with 70% ethyl alcohol and a 1–2% povidone iodine solution was performed before BC collection in patients with suspected sepsis. Two or three BC samples per patient, collected 5–30 min apart, were obtained from two different anatomical sites. BC bottles were sent to the Microbiology Laboratory as soon as possible (within 2 h), and an automated BC system (BACTEC 9240, BD, Becton Dickinson, Istanbul, Turkiye) was used for analysis.

Subcultures were prepared from bottles that yielded a positive signal, plated onto 5% sheep blood agar, and incubated at 35 °C for 18–24 h. Gram staining was performed on the growing colonies, and bacterial identification and antibiotic susceptibility tests were performed by transferring them to the relevant automated identification panel (BD Phoenix, Beckton Dickinson, Istanbul, Turkiye). The recommendations of the European Committee for Antimicrobial Susceptibility Testing (EUCAST) were used for antibiotic susceptibility testing [26].

### 4.6. rRT-PCR Method

**Figure 1:**
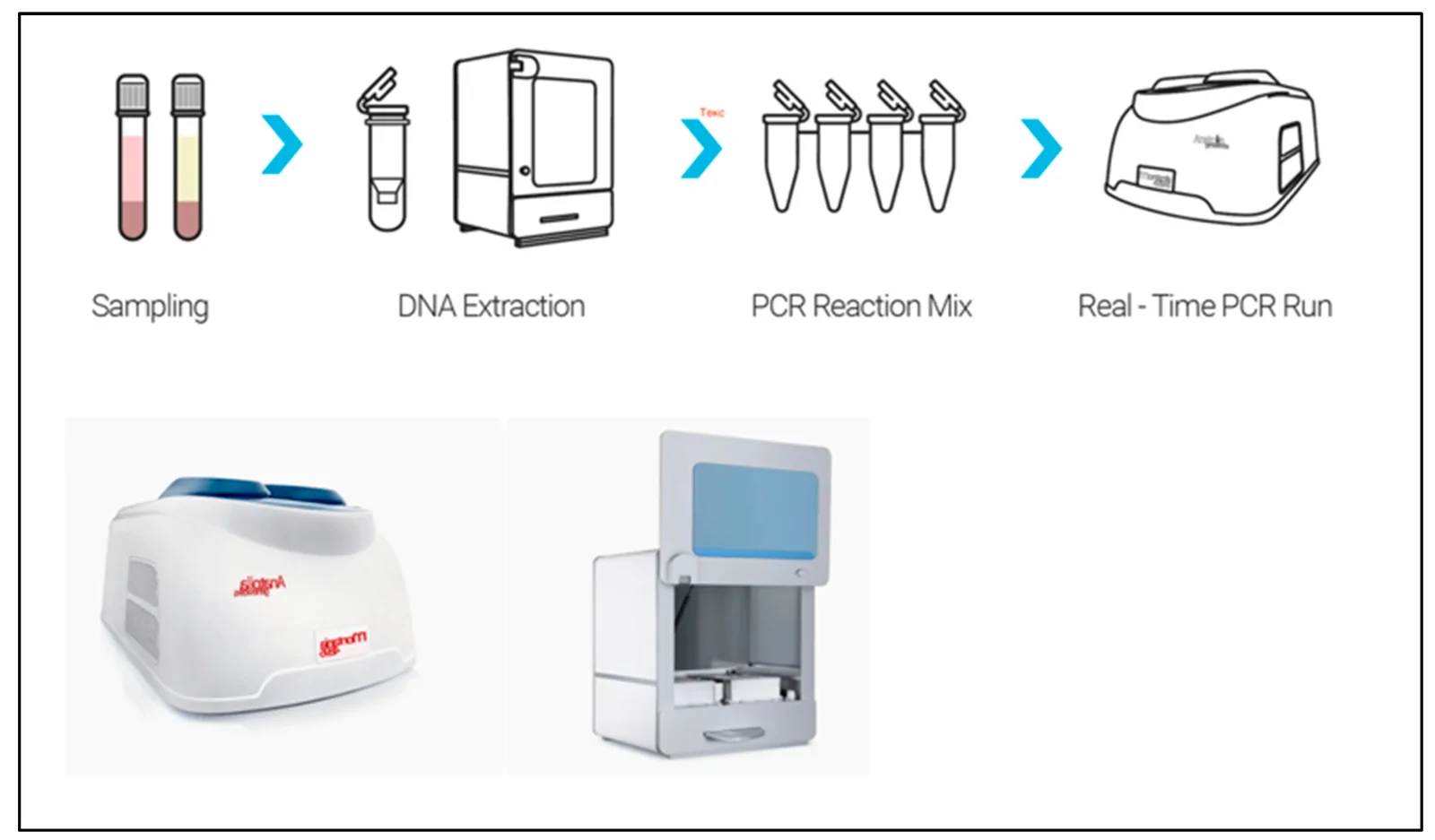
Flow chart of the rRT-PCR system from directly whole blood.

Molecular testing was performed using whole-blood samples according to the manufacturer’s instructions (Figure 1). Briefly, whole-blood samples were processed on the UNIO B24 Extraction System (Anatolia Geneworks, Istanbul, Turkiye; Cat. No. AUB24) using the UNIO Whole Blood Genomic DNA Extraction Large Volume Kit (Anatolia Geneworks, Istanbul, Turkiye; Cat. No. UGDL600). The input volume was 600 µL, and the final elution volume was 150 µL. The turnaround time from sample processing to the first molecular result was approximately four hours.

qPCR amplification was performed using the Bosphore Sepsis Panel Bundle Kit (Anatolia Geneworks, Istanbul, Turkiye; Cat. No. ABSPB2), which included the Bosphore Sepsis Screening Kit and the Bosphore ABR Screening Kit, according to the manufacturer’s instructions. The assay is based on a multiplex qPCR approach, allowing multiple targets to be detected simultaneously within each PCR master mix using different fluorescence channels. The rRT-PCR panel targets 32 pathogens, namely *Staphylococcus* spp., *Staphylococcus aureus*, *Staphylococcus epidermidis*, *Streptococcus pneumoniae*, *Streptococcus* spp., *Streptococcus pyogenes*, *Streptococcus agalactiae*, *Enterococcus faecalis*, *Enterococcus faecium*, *Listeria monocytogenes*, *Escherichia coli*, *Enterobacter cloacae*, *Klebsiella pneumoniae*, *Klebsiella oxytoca*, *Klebsiella aerogenes*, *Proteus mirabilis*, *Salmonella enterica*, *Serratia marcescens*, *Campylobacter* spp., *Acinetobacter baumannii*, *Pseudomonas aeruginosa*, *Stenotrophomonas maltophilia*, *Haemophilus influenzae* (type b), *Neisseria meningitidis*, *Candida albicans Candida tropicalis*, *Candida glabrata*, *Candida parapsilosis*, *Candida krusei*, *Candida auris*, *Aspergillus* spp., and *Fusarium* spp. The antimicrobial resistance marker panel targets OXA-48, NDM, KPC, IMP, VIM, CTX-M, *mcr*-1, *van*A, *van*B, and *mec*A resistance genes. The kit also includes an internal control to monitor DNA extraction, PCR inhibition, and process-related errors. Additionally, real-time PCR was performed using the Montania 4896 Real-Time PCR Instrument (Anatolia Geneworks, Istanbul, Turkiye).

### 4.7. Statistical Analysis

All statistical analyses were conducted with IBM SPSS Statistics version 26.0 (IBM Statistics, İstanbul, Türkiye). Descriptive statistics were employed to summarize the variables. Categorical data were reported as numbers and percentages, whereas continuous variables were presented as median values along with their range (min–max). The Kolmogorov–Smirnov test was used to evaluate data distribution, and the association between categorical variables was evaluated using Pearson’s chi-square or Fisher’s exact test. Continuous data from two independent groups were analyzed using the Mann–Whitney U test, and results with *p* < 0.05 were considered statistically significant. Cohen’s Kappa test was used to analyze the agreement between the two methods, and the diagnostic performance of the rRT-PCR method was evaluated by calculating sensitivity, specificity, positive predictive value (PPV), negative predictive value (NPV) and accuracy and compared against the reference method.

## 5. Conclusions

The results of the present study indicate that the rRT-PCR kit, used within an automated extraction and multiplex qPCR-based workflow, can be implemented for direct whole-blood molecular testing in patients with suspected clinical sepsis. This workflow may support earlier pathogen and resistance-marker detection without waiting for culture positivity. In addition, the availability of results within four hours represents an important practical advantage in critically ill or clinically unstable patients, where earlier microbiological information may support timely therapeutic decision-making.

Therefore, when interpreted together with clinical findings and blood culture results, this automated extraction and multiplex qPCR-based workflow may serve as a rapid adjunctive diagnostic tool for earlier pathogen identification, resistance-marker detection, and antimicrobial stewardship, as well as timely optimization of treatment, in suspected bloodstream infections. While blood cultures should continue to be performed for viable organism recovery and phenotypic antimicrobial susceptibility testing, multiplex qPCR may provide earlier actionable microbiological information, thereby allowing clinicians to reassess empirical therapy before conventional culture-based results are finalized. Future studies guided by rRT-PCR should focus on clinical outcomes such as antimicrobial management, mortality rates, length of hospital stay, and cost-effectiveness.

## Figures and Tables

**Table 1:** Demographic, clinical, and laboratory parameters of the study population.

Demographic Characteristics	Min–Max	Median	Mean-Std. Dev.	n	%
Age	19–92	63	59.1 ± 17.8		
Gender					
Female				50	31.3
Male				110	68.8
SIRS score	1–17	4	4.4 ± 2.3		
APACHE score	2–37	14	14.4 ± 8.3		
SOFA score	1–15	4.5	5.0 ± 3.5		
ASA score					
I				34	21.3
II				90	56.3
III				30	18.8
IV				6	3.8
WBC	10–141,000	9950	12,466.8 ± 13,693.6		
HGB	4–27	9	9.4 ± 3.0		
HTC	7–91	28	28.1 ± 8.2		
CRP	0.5–554	139	154.2 ± 105.7		
PCT	0–241	1	11.7 ± 29.7		
Bilirubin	0–34	062	1.31 ± 3.43		
Lactate	0.3–20	1	1.99 ± 3.08		
ALT	0–16,000	26	208.8 ± 1322.4		
AST	0–18,000	28.5	285.7 ± 1539.7		
Creatinine	0.1–7	0.9	1.07 ± 0.97		
Comorbidity					
No				4	2.5
Yes				156	97.5
Hypertension				40	25
Solid malignity				32	20
Hematological malignity				28	17.5
Diabetes mellitus				12	7.5
Chronic lung disease				11	6.9
Chronic renal failure				11	6.9
Rheumatological disease				6	3.8
Hepatocellular carcinoma				4	2.5
COPD ^1^				3	1.9
Infectious disease				2	1.3
Ulcerative colitis				2	1.3
Others				5	3.1
Hospital Wards					
Intensive Care Units				99	61.9
Emergency Units				39	24.4
Other wards				22	13.8
30 days mortality					
No				114	71.3
Yes				46	28.7

^1^ Chronic obstructive pulmonary disease.

**Table 2:** Distribution of microorganisms detected by BC and rRT-PCR.

Microorganisms Growth	Blood Culture		rRT-PCR	
	n	%	n	%
No growth	106	66.25	118	73.75
**Gram-positive bacteria**				
*Staphylococcus* spp.			9	5.625
*Staphylococcus aureus*	10	6.25	4	2.5
Methicillin resistant—coagulase negative *Staphylococcus* (MRCNS)	3	1.87		
Methicillin susceptible—coagulase negative *Staphylococcus* (MSCNS)	2	1.25		
*Enterococcus faecium*	3	1.87	3	1.87
*Streptococcus* spp.	2	1.25	2	1.25
**Gram-negative bacteria**				
*Escherichia coli* (ESBL-negative)	5	3.12	3	1.87
*Escherichia coli* (ESBL-positive)	3	1.87	4	2.5
*Klebsiella pneumoniae* (ESBL-negative)	4	2.5	5	3.12
*Klebsiella pneumoniae* (ESBL-positive)	6	3.75	6	3.75
*Pseudomonas aeruginosa*	1	0.62		
*Acinetobacter* spp.	1	0.62		
*Enterobacter* spp.			1	0.62
*Haemophilus influenzae*	1	0.62		
*Morganella morganii*	2	1.25		
**Fungi**				
*Candida* spp.	2	1.25		
*Candida albicans*	3	1.87	3	1.87
*Candida parapsilosis*	2	1.25		
*Candida tropicalis*	2	1.25	2	1.25
*Cryptococcus* spp.	2	1.25		
Total	160	100	160	100
Concordance between two methods (Cohen’s Kappa)	127	79.4	*p* = 0.000 (95% CI)	

**Table 3:** Comparison of demographic characteristics, comorbidities, and clinical severity scores according to BC and rRT-PCR concordance.

Demographic Characteristics Comorbidities Clinical Severity Scores Hospital Wards Positive Signal Time	Blood Culture–rRT-PCR Concordance				
	Incompatible Group (n = 33)		Compatible Group (n = 127)		
	Mean-Std. Dev. n/%	Median	Mean-Std. Dev. n/%	Median	*p* Value
Age	53 ± 18.2	56	60.7 ± 17.4	63	**0.029** *
Gender					0.477 **
Female	12/36.4		38/29.9		
Male	21/63.6		89/70.1		
SIRS score	4.8 ± 1.9	5	4.6 ± 2.3	4	0.093 **
APACHE score	15.8 ± 8.0	15	14.1 ± 8.3	12	0.224 **
SOFA score	5.6 ± 3.3	6	4.9 ± 3.5	4	0.130 **
ASA score					0.788 **
I	7/21.2		27/21.3		
II	18/54.5		72/56.7		
III	7/21.2		23/18.1		
IV	1/3		5/3.9		
Comorbidity					0.582 **
No	0/0		4/3.1		
Yes	33/100		123/96.9		
Hypertension	7/21.2		33/26.8		0.573 **
Solid malignity	5/15.2		27/22		0.434 **
Hematological malignity	9/27.3		19/15.4		0.097 **
Diabetes mellitus	3/9.1		9/7.3		0.697 **
Chronic lung disease	1/3		10/8.1		0.327 **
Chronic renal failure	2/6.1		9/7.3		0.836 **
Rheumatological disease	2/6.1		4/3.3		0.604 **
Hepatocellular carcinoma	0/0		4/3.3		0.582 **
COPD ^1^	2/6.1		1/0.8		0.108 **
Infectious disease	0/0		2/1.06		1.000 **
Ulcerative colitis	1/3		1/0.8		0.371 **
Others	1/3		4/3.3		1.000 **
Hospital Wards					0.648 **
Intensive Care Units	22/66.7		77/60.6		
Emergency Units	6/18.2		33/26		
Other wards	5/15.2		17/13.4		
Positive signal time (h)	26.4 ± 18.1		6.4 ± 14.8		**0.001** **

^1^ Chronic obstructive pulmonary disease. * Mann-Whitney U test; ** χ^2^ test.

**Table 4:** The comparison of pathogens detected by BC and rRT-PCR.

	rRT-PCR																				
	No detection	*S. aureus*	Met-susceptible-CNS	Met-resistant-CNS	*K. pneumoniae* ESBL (−)	*E. coli* ESBL (−)	*Candida* spp.	*C. albicans*	*C. parapsilosis*	*C. tropicalis*	*Acinetobacter* spp.	*M. morganii*	*P. aeruginosa*	*H. influenzae*	*Streptococcus* spp.	*E. faecium*	*Cryptococcus* spp.	*E. coli* ESBL (+)	*K. pneumoniae* ESBL (+)	*Staphylococcus* spp.	*Enterobacter* spp.
**BLOOD CULTURE**																					
No growth	**100**	1	0	0	1	0	0	1	0	0	0	0	0	0	1	0	0	1	0	1	0
*S. aureus*	1	**3**	0	0	0	0	0	0	0	0	0	0	0	0	0	0	0	0	0	6	0
Met.-susceptible-CNS	2	0	**0**	0	0	0	0	0	0	0	0	0	0	0	0	0	0	0	0	0	0
Met.-resistant-CNS	1	0	0	**0**	0	0	0	0	0	0	0	0	0	0	0	0	0	0	0	2	0
*K.pneumoniae* ESBL (−)	0	0	0	0	**4**	0	0	0	0	0	0	0	0	0	0	0	0	0	0	0	0
*E. coli-* ESBL (−)	1	0	0	0	0	**3**	0	0	0	0	0	0	0	0	0	0	0	0	0	0	1
*Candida* spp.	2	0	0	0	0	0	**0**	0	0	0	0	0	0	0	0	0	0	0	0	0	0
*C.albicans*	1	0	0	0	0	0	0	**2**	0	0	0	0	0	0	0	0	0	0	0	0	0
*Candida parapsilosis*	2	0	0	0	0	0	0	0	**0**	0	0	0	0	0	0	0	0	0	0	0	0
*C. tropicalis*	0	0	0	0	0	0	0	0	0	**2**	0	0	0	0	0	0	0	0	0	0	0
*Acinetobacter* spp.	1	0	0	0	0	0	0	0	0	0	**0**	0	0	0	0	0	0	0	0	0	0
*M. morganii*	2	0	0	0	0	0	0	0	0	0	0	**0**	0	0	0	0	0	0	0	0	0
*P. aeruginosa*	1	0	0	0	0	0	0	0	0	0	0	0	**0**	0	0	0	0	0	0	0	0
*H. influenzae*	1	0	0	0	0	0	0	0	0	0	0	0	0	**0**	0	0	0	0	0	0	0
*Streptococcus* spp.	1	0	0	0	0	0	0	0	0	0	0	0	0	0	**1**	0	0	0	0	0	0
*E. faecium*	0	0	0	0	0	0	0	0	0	0	0	0	0	0	0	**3**	0	0	0	0	0
*Cryptococcus* spp.	2	0	0	0	0	0	0	0	0	0	0	0	0	0	0	0	**0**	0	0	0	0
*E. coli* ESBL (+)	0	0	0	0	0	0	0	0	0	0	0	0	0	0	0	0	0	**3**	0	0	0
*K. pneumoniae* ESBL (+)	0	0	0	0	0	0	0	0	0	0	0	0	0	0	0	0	0	0	**6**	0	0
*Staphylococcus* spp.	0	0	0	0	0	0	0	0	0	0	0	0	0	0	0	0	0	0	0	**0**	0
*Enterobacter* spp.	0	0	0	0	0	0	0	0	0	0	0	0	0	0	0	0	0	0	0	0	**0**

**Table 5:** Comparison of microbiological results based on BC-rRT-PCR concordance.

Comparison of Microbiological Results Based BC-rRT-PCR Concordance	Blood Culture–rRT-PCR Concordance				
	Incompatible Group (n = 33)		Compatible Group (n = 127)		
	Mean-Std. Dev. n/%	Median	Mean-Std. Dev. n/%	Median	*p* Value
Positive signal time (BC)	26.4 ± 18.1	24	6.4 ± 14.8	0	**0.000** *
How many BC bottles growth					0.060 **
I	10/37		2/7.4		
II	6/22.2		7/25.9		
III	1/3.7		3/11.1		
IV	10//37		15//55.6		
2. causative agent in BC					0.371 **
No growth	32/97		126/99.2		
*K. pneumoniae*	1/3		1/0.8		
In how many vials did the second agent grow?					1.000 **
II	0/0		1/100		
IV	1/100		0/0		
2. causative agent using rRT-PCR					0.108 **
No detected	31/93.9		126/99.2		
*K. pneumoniae*	2/6.1		1/0.8		
Resistance genes detected using rRT-PCR					0.258 **
No	29/87.9		119/93.7		
Yes	4/12.1		8/6.3		
Growth in other specimens					**0.002** **
No	17/51.5		100/78.7		
Yes	16/48.5		27/21.3		

* Mann-Whitney U test; ** χ^2^ test.

**Table 6:** Comparison of demographic characteristics, comorbidities, and clinical severity scores according to 30-day mortality status.

	30-Day Mortality Status				
	30-Day Mortality (−) (n = 114)		30-Day Mortality (+) (n = 46)		
	Mean-Std. Dev. n/%	Median	Mean-Std. Dev. n/%	Median	*p* Value
Age	58 ± 17.4	62.5	61.8 ± 18.5	66.5	0.157 *
Gender					0.888 **
Female					
Male					
SIRS score	4.1 ± 2.2	4	5.2 ± 2.1	6	**0.001** **
APACHE score	12.6 ± 7.4	12	18.9 ± 8.7	19	**0.000** **
SOFA score	4.4 ± 3.1	4	6.7 ± 3.9	6	**0.000** **
ASA score					**0.005** **
I	28/24.6		6/13		
II	67/58.8		23/50		
III	18/15.8		12/26.1		
IV	1/0.9		5/10.9		
Comorbidity					0.579
No	4	3.5	0	0	
Yes	110	96.5	46	100	
Hypertension	22	20	18	39.1	**0.009** **
Solid malignity	21	19.1	11	23.9	0.432
Hematological malignity	19	17.3	9	19.6	0.662
Diabetes mellitus	11	10	1	2.2	0.104
Chronic lung disease	8	7.3	3	6.5	0.911
Chronic renal failure	10	9.1	1	2.2	0.135
Rheumatological disease	5	4.5	1	2.2	0.674
Hepatocellular carcinoma	3	2.7	1	2.2	1.000
COPD ^1^	2	1.8	1	2.2	1.000
Infectious disease	2	.18	0	0	1.000
Ulcerative colitis	2	1.8	0	0	1.000
Others	5	4.5	0	0	0.322
Hospital Wards					**0.007** **
Intensive Care Units	62/54.4		37/80.4		
Emergency Units	32/28.1		7/15.2		
Other wards	20/17.5		2/4.3		

^1^ Chronic obstructive pulmonary disease. * Mann-Whitney U test; ** χ^2^ test.

**Table 7:** Comparison of surgical history and antimicrobial treatment in patient groups with and without 30-day mortality.

	30-Day Mortality Status		
	30-Day Mortality (−) (n = 114)	30-Day Mortality (+) (n = 46)	
	n/%	n/%	*p* Value
History of Previous Surgery			0.424 *
No	60/52.6	21/45.7	
Yes	54/47.4	25/54.3	
Medication Use			0.741 *
No	25/21.9	9/19.6	
Yes	89/78.1	37/80.4	
History of Antibiotic Use in the Last 3 Months			0.751 *
No	70/61.4	27/58.7	
Yes	44/38.6	19/41.3	
Antibiotic Use			1.000 *
No	2/1.8	0/0	
Yes	112/98.2	46/100	
2nd Antibiotic Use			**0.002** *
No	61/53.5	12/26.1	
Yes	53/46.5	34/73.9	
3rd Antibiotic Use			0.235 *
No	102/89.5	38/82.6	
Yes	12/10 5	8/17.4	

* Mann-Whitney U test.

**Table 8:** Comparison of biochemical parameters in the 30-day mortality and non-mortality groups.

	30-Day Mortality Status				
	30-Day Mortality (−) (n = 114)		30-Day Mortality (+) (n = 46)		
	Mean-Std Dev.	Median	Mean-Std dev.	Median	*p* Value
WBC	13,145 ± 15,056	10,000	10,787 ± 9448	8550	0.298 *
HGB	9.47 ± 3.01	9.00	9.11 ± 3.09	9.00	0.157 *
HTC	28.8 ± 8.8	28.0	26.5 ± 6.3	27.0	0.102 *
CRP	152.3 ± 106.6	139.0	158.7 ± 104.5	137.0	0.716 *
PCT	9.88 ± 23.12	1.00	16.33 ± 41.68	3.00	**0.049** *
Bilirubin	1.05 ± 3.34	0.53	1.96 ± 3.59	0.96	**0.000** *
Lactate	1.82 ± 2.66	1.00	2.40 ± 3.93	1.00	0.902 *
ALT	131.5 ± 481.6	24.5	400.4 ± 2354.4	27.0	0.529 *
AST	213.4 ± 1685.7	25.0	465.0 ± 1093.1	60.0	**0.000** *
Creatinine	1.05 ± 0.86	0.90	1.13 ± 1.22	0.89	0.905 *

* Mann-Whitney U test.

**Table 9:** Diagnostic performance of the rRT-PCR method compared to the reference BC method.

**Rapid-RT-PCR**		**Blood Culture (Reference Method)**		
		**Positive**	**Negative**	**Total**
	Positive	46 (TP)	1 (FP)	47
	Negative	11 (FN)	102 (TN)	113
	Total	57	103	160
Sensitivity	TP/(TP + FN) × 100			80.7%
Specificity	TN/(TN + FP) × 100			99.0%
Accuracy	TP + TN/(TP + TN + FP + FN) × 100			92.5%
PPV	TP/(TP + FP) × 100			97.8%
NPV	TN/(TN + FN) × 100			90.2%

True positives, TN: True negatives, FP: False positives, FN: False negatives, PPV: Positive predictive value, NPV: Negative predictive value.

## Data Availability

The data presented in this study are available on request from the corresponding author due to confidentiality and ethical constraints.

## Abbreviations

The following abbreviations are used in this manuscript:

AST	Aspartate amino transferase
APACHE	Acute Physiology and Chronic Health Evaluation
ARDS	Acute Respiratory Disease Syndrome
BC	Blood Culture
CRP	C-Reactive Protein
ESBL	Extended Spectrum-Beta Lactamase
EUCAST	The European Committee on Antimicrobial Susceptibility Testing
FN	False negatives
FP	False positives
GCS	Glascow Coma Score
HGB	Hemoglobin
HTC	Hematocrit
IMP	Imipenemase
ICU	Intensive Care Unit
KPC	Klebsiella pneumoniae Carbapenemase
MDR	Multiple Drug-Resistance
NDM	New Delhi Metallo-beta lactamase
NPD	Negative Predictive Value
PCT	Procalcitonin
PPD	Positive Predictive Value
SIRS	Systemic Inflammatory Response Syndrome
SOFA	Sequential Organ Failure Assessment Score
SPSS	Statistical Package for the Social Sciences
TN	True negatives
TP	True positives
